# Transglutaminase 2 regulates ovarian cancer metastasis by modulating the immune microenvironment

**DOI:** 10.3389/fimmu.2025.1639853

**Published:** 2025-07-24

**Authors:** Dalia Ibrahim, Melanie Grondin, Kristianne Galpin, Sara Asif, Emily Thompson, Sarah Nersesian, John Abou-Hamad, Maryam Echaibi, Galaxia M. Rodriguez, Pauline Navals, Elizabeth Macdonald, Brianna Ryan, David P. Cook, Jeffrey W. Keillor, Barbara C. Vanderhyden

**Affiliations:** ^1^ Cancer Research Program, Ottawa Hospital Research Institute, Ottawa, ON, Canada; ^2^ Department of Cellular and Molecular Medicine, University of Ottawa, Ottawa, ON, Canada; ^3^ Department of Microbiology and Immunology, University of Ottawa, Ottawa, ON, Canada; ^4^ Department of Chemistry and Biomolecular Sciences, University of Ottawa, Ottawa, ON, Canada

**Keywords:** ovarian cancer, transglutaminase 2, immune modulation, targeted therapy, metastasis

## Abstract

**Introduction:**

Ovarian cancer is the most lethal gynecological malignancy. Deepening our knowledge of the interactions within the tumor microenvironment (TME) is important for discovering new targeted treatment strategies. Transglutaminase 2 (TG2) is a protein implicated in many biological and pathophysiological processes, including promoting tumor progression in ovarian cancer. Its role in disease progression has been studied in ovarian cancer cells; however, its role in the ovarian TME is less understood.

**Methods:**

In this study, for the first time, we assessed the therapeutic potential of novel covalent irreversible small molecule TG2 inhibitors in xenograft models of ovarian cancer. We further elucidated the role of TG2 in ovarian cancer cells and syngeneic tumors by immune phenotyping using flow cytometry, RNA sequencing, and immunohistochemistry to characterize the contribution of TG2 in the TME to the metastatic process of ovarian cancer.

**Results:**

To investigate the transamidation catalytic and GTP binding activities of TG2 in cancer cells, we used several TG2 inhibitors, some of which decreased invasiveness of human ovarian cancer cell lines in vitro and lengthened survival of the SKOV3 xenograft model. Using the ID8 *Trp53^-/-^ Brca1^-/-^* and KPCA.B syngeneic mouse models of ovarian cancer, we defined the contribution of TG2 in the TME to the metastatic process. Lack of TG2 in the TME prolonged survival in the ID8 *Trp53^-/-^ Brca1^-/-^* metastatic model, but it did not affect survival in the non-metastatic KPCA.B model. Through extensive analysis of the immune composition in both the primary tumor and metastatic ascites in the ID8 *Trp53^-/-^ Brca1^-/-^* model, we discovered that the lack of host TG2 resulted in decreased frequency of immunosuppressive tumor-associated macrophages, and increased frequency of T cells, NK cells, and B cells. RNA sequencing of the primary tumors with or without TG2 present in the TME, revealed an enrichment of pathways related to B cell activation and regulation.

**Discussion:**

These findings highlight the importance of TG2 in the TME for ovarian cancer metastasis, potentially by activation of humoral immunity and specifically highlight a crucial role for TG2 in modulating B cells to prolong survival in mouse models of ovarian cancer.

## Introduction

Ovarian cancer is the most lethal gynecological malignancy with a poor 5-year survival of 44% (1). The complex interactions between cancer cells and their surrounding tumor microenvironment (TME) can lead to immunosuppression and the development of chemoresistance, which has made the development of effective therapeutic strategies challenging (2). This emphasizes the importance of furthering our knowledge of this interplay to identify new therapeutic targets. Thus, the ovarian cancer field has focused heavily on understanding the TME and the role of non-cancerous cells in modulating disease progression. Studies have outlined the role of immune cells including dendritic cells, natural killer (NK) cells, B cells, T cells, and tumor-associated macrophages (TAMs) in promoting ovarian cancer tumorigenesis and metastatic dissemination through immunosuppression (3–5). As a result, immune checkpoint inhibitors have been tested in clinical trials as monotherapies or in combination with chemotherapy. However, outcomes have not been positive, in part due to the immunosuppression caused by an abundance of cell types and their complex interactions within the TME (6–9). More extensive research into the factors that may regulate the presence or activity of the various cell types in the TME is crucial to overcome these barriers.

Transglutaminase 2 (TG2) is a multifunctional protein with conformationally mutually exclusive transamidation catalytic activity and GTP binding activity (10). Beyond these two main functions, TG2 is also known to independently form a protein-protein complex with fibronectin. In many epithelial ovarian cancers, TG2 is highly expressed compared to normal ovarian surface epithelium, and plays an important role in promoting disease progression by increasing ovarian cancer cell dissemination and accelerating peritoneal disease through its various functions (11, 12). Specifically, elevated TG2 expression has been associated with a more migratory and invasive phenotype, due in part to its role in promoting an epithelial-to-mesenchymal transition (EMT) (13). Many studies on TG2 have focused on elucidating its role in cancer cells; however, TG2 also regulates the immune system in various types of cancers (14). High expression of TG2 in macrophages is associated with a more immunosuppressive phenotype (M2 polarized) in asthma (15). It also affects the interaction between dendritic cells and T cells to decrease anti-tumoral T cell function (16). In a syngeneic model of ovarian cancer, absence of host TG2 was found to promote CD8+ T cell cytotoxic activity (17). Further exploring its role and impact on the immune system could help establish if targeting TG2 could be beneficial as a treatment to enhance anti-tumor immunity in ovarian cancer.

As TG2 is implicated in many pathophysiological processes, developing strategies that target this protein by inhibiting its activity is critical. Many TG2 inhibitors, reversible and irreversible, have been developed (18). NC9 and VA4 are irreversible small molecule TG2 inhibitors that bind covalently to the catalytic site of TG2 resulting in the abolition of the enzyme’s conformational dynamism, and therefore abolishing both its catalytic and GTPase activities (19–21). Previous studies have demonstrated the efficacy of NC9 and VA4 both *in vivo* and *in vitro* in other pathologic conditions including mesothelioma, squamous cell carcinoma, and recovery from spinal cord injury (22–24). This study is the first to test these inhibitors in xenograft models of ovarian cancer to assess their therapeutic potential and to evaluate the role of TG2 transamidation and GTP binding functions in disease progression.

In this study, we conducted a comprehensive characterization of the functions of TG2 and the relative contribution of TG2 in ovarian cancer cells and the TME to ovarian cancer disease progression. Treatment of the ovarian cancer cells with TG2 inhibitors decreased their invasive phenotype. However, *in vivo* application of TG2 inhibitors yielded only a modest increase in survival in a xenograft model which led us to investigate the role of TG2 in the TME. Syngeneic orthotopic transplant of ovarian cancer cells into transgenic mice lacking TG2 resulted in delayed metastasis, prolonged survival, and notable alterations in anti-tumor immune composition in mice harboring ID8 *Trp53^-/-^ Brca1^-/-^* tumors, highlighting the importance of TG2 in the TME for disease progression.

## Results

### TG2 shows variable expression in human ovarian cancer cell lines and TG2 inhibitors reduce cell invasive capacity

**Figure 1:**
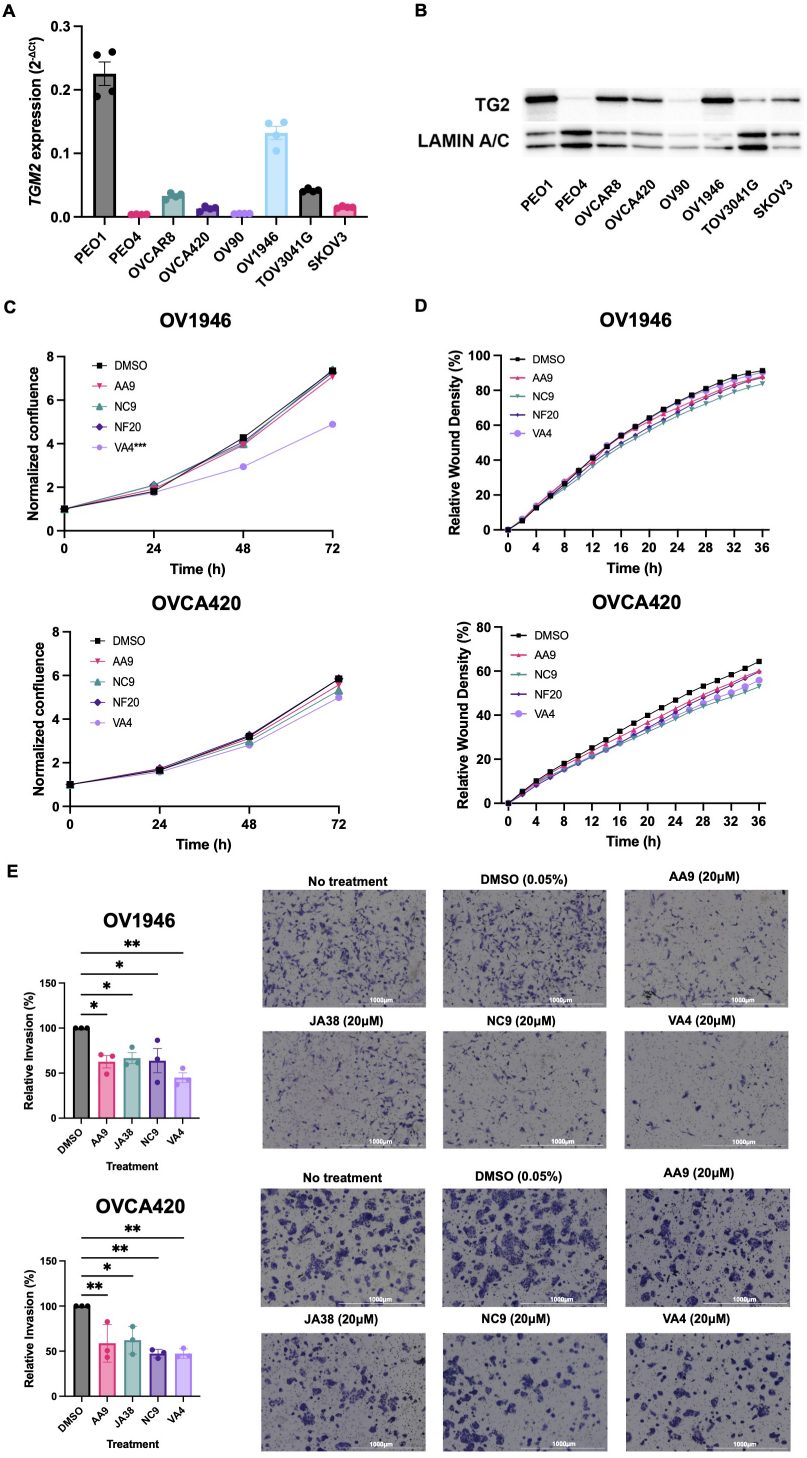
TG2 inhibitors primarily affect cell invasive capacity of OV1946 and OVCA420 human ovarian cancer cell lines. **(A)** Baseline *TGM2* expression in eight human ovarian cancer cell lines measured by qPCR. Expression was determined using the delta CT method and was normalized to the housekeeping gene *PPIA*. Values shown are the mean ± SEM (n=4 per cell line). **(B)** TG2 protein abundance in eight human ovarian cancer cell lines relative to the loading control Lamin A/C. **(C)** OV1946 and OVCA420 cell lines were treated with TG2 inhibitors AA9, NC9, NF20, VA4 at a concentration of 20μM or with an equivalent volume of DMSO as a vehicle control. Proliferation was assessed based on cell confluency using Incucyte. Data were normalized to confluence at the 0 h time point. Values shown are the mean (n=3 per experimental group). Significant differences were determined by repeated measure two-way ANOVA. ***p<0.001. **(D)** Migration was assessed by scratch-wound assay of cells treated with TG2 inhibitors at a concentration of 20μM and cell density in the wound over time was recorded by Incucyte. Values shown are the mean (n=3 per experimental group). Significant differences were determined by repeated measure two-way ANOVA. **(E)** Invasion was assessed by Transwell assays with TG2 inhibitors AA9, JA38, NC9, and VA4 at a concentration of 20μM. Data were normalized to the DMSO control. Images show the cells that invaded with each treatment. Values shown are the mean ± SEM (n=3 per experimental group). Significant differences were determined by one-way ANOVA and Dunnett’s *post-hoc* test. *p<0.05, **p<0.01.

TG2 is overexpressed in many epithelial ovarian cancers relative to normal ovarian surface epithelium, and its expression is associated with enhanced metastasis and poorer prognosis (13, 25). To identify cell lines that express TG2, we quantified TG2 by qPCR and western blot analysis in a panel of eight human ovarian cancer cell lines, finding it variable but with good consistency between transcript and protein levels for each cell line except for the TOV3041G cell line which has less TG2 protein expression than is predicted by the mRNA transcript levels, potentially due to intrinsic differences in translational regulation (**Figures 1A, B**). Previous studies have found that TG2 promotes cell survival, proliferation, migration, and invasive phenotypes (26). We selected four cell lines that have variable levels of TG2 expression (OV1946, OVCA420, TOV3041G and SKOV3) to further elucidate the role of TG2 in these pathophysiological processes. To test the influence of TG2 on phenotype, we used irreversible TG2 inhibitors AA9, NC9, NF20, JA38, and VA4, which bind to the transamidation catalytic site of TG2, abolishing both its transamidation catalytic and GTP binding activities while leaving the fibronectin-binding function intact (19, 20, 27–29). In the high-TG2 OV1946 cell line, the VA4 inhibitor reduced cell proliferation while no inhibitors affected cell proliferation in the OVCA420 cells (**Figure 1C**). There was also no impact on cell migration in a wound closure assay compared to the DMSO control in either cell line (**Figure 1D**). These results were consistent with the results of similar assays using TOV3041G and SKOV3 cell lines (**Supplementary Figure S1**). The invasive capacity of both the OV1946 and OVCA420 cell lines was significantly decreased by all four TG2 inhibitors (AA9, JA38, NC9, and VA4) (**Figure 1E**) (20). This indicates that while the transamidation catalytic functions and GTP binding activities of TG2 do not influence proliferation and migration in these cell lines, they may be necessary for promoting cell invasion.

### The TG2 inhibitor VA4 prolongs survival in SKOV3 tumor-bearing mice

**Figure 2:**
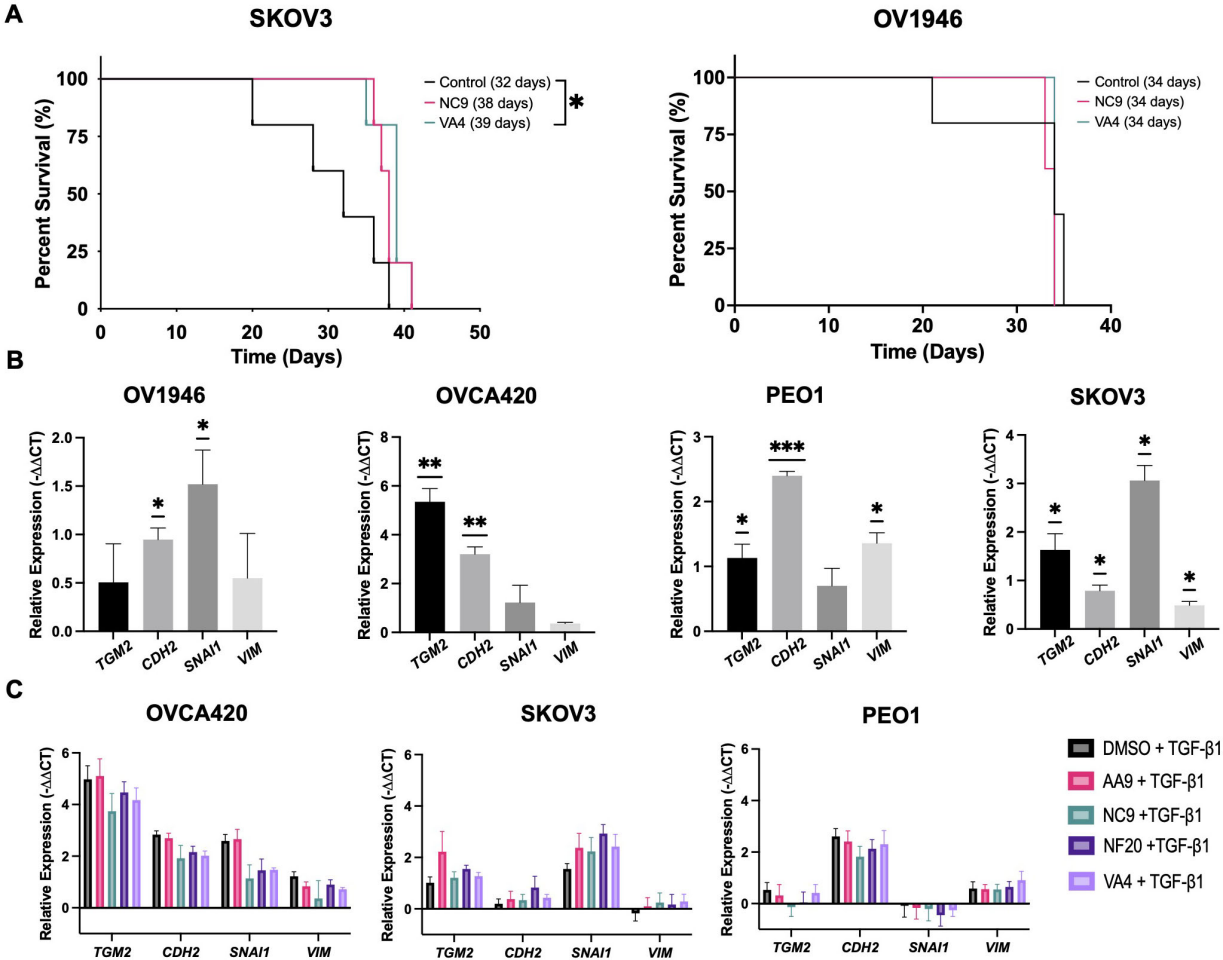
TG2 inhibitors tested herein do not affect the expression of EMT markers in ovarian cancer cell lines but modestly prolong survival in the SKOV3 xenograft model of ovarian cancer. **(A)** SKOV3 cells (left) or OV1946 cells (right) were injected intraperitoneally into NOD *scid* gamma mice (n=3–5 per experimental group). Starting at 25% of the median survival known for these models, mice were treated with either NC9 (20 mg/kg), VA4 (20 mg/kg), or DMSO as a vehicle control three times per week until humane endpoint. Statistically significant differences were determined by Log-rank (Mantel-Cox) test. *p<0.05. **(B)** Cells were treated with TGF-β1 (10 ng/ml) for 48 hours and expression of *TGM2* and the EMT genes *CDH2, SNAI1, and VIM* was assessed by qPCR. Values shown are the mean ± SEM (n=3 per experimental group). Statistical significances were determined by one-sample t and Wilcoxon test. *p<0.05, **p<0.01, ***p<0.001 **(C)** Cells were treated with TG2 inhibitors AA9, NC9, NF20, VA4 (20μM) or DMSO as a vehicle control prior to TGF-β1 (10 ng/mL) treatment for 48 hours. Expression of *TGM2* and the EMT genes *CDH2, SNAI1, and VIM* was assessed by qPCR. Values shown are the mean ± SEM (n=3 per experimental group). Significant differences were determined by one-way ANOVA and Dunnett’s *post-hoc* test.

Since the VA4 TG2 inhibitor decreased cell invasion in the OV1946 cells and these cells have previously been used to develop a xenograft model (30), we assessed whether the TG2 inhibitors could prolong survival in the OV1946 and SKOV3 ovarian cancer xenograft models. SKOV3 (low TG2 expression) and OV1946 (high TG2 expression) cancer cells were injected intraperitoneally into NOD *scid* gamma (NSG) mice. The mice were then treated with NC9, VA4, or DMSO vehicle control as these inhibitors have been previously shown to elicit positive responses in xenograft models of other malignancies (22, 24). VA4 treatment prolonged survival by 22% in SKOV3-bearing mice, which survived a median of 39 days, compared to 32 days in the DMSO vehicle control (**Figure 2A**). No difference was observed in OV1946 tumor-bearing mice with either TG2 inhibitor (**Figure 2A**). These observations suggest that blocking the TG2 catalytic and GTPase activities in the OV1946 model may not be sufficient to delay tumor progression but may be sufficient for delaying tumor progression to a modest extent in the SKOV3 model.

### The TG2 inhibitors tested do not affect the expression of EMT genes

Since VA4 delayed tumor progression in the SKOV3 model, we then assessed if this was through the EMT. EMT is a key process by which cancer cells develop an invasive phenotype, and it is well established that TG2 plays a role in the EMT process (13, 31). Previous RNA-sequencing results from TGF-β1-treated OVCA420 cells showed *TGM2* to be highly upregulated along with other canonical markers of EMT (*CD44*, *TGFβ1*, and *FN1*) (32). *TGM2* expression was also increased in MCF7 (breast cancer), DU145 (prostate cancer), and A549 (lung cancer) cell lines in response to two other EMT-inducers TNFα and EGF, demonstrating that increased *TGM2* expression is consistently associated with the EMT process (32). We therefore determined if the process by which the inhibitors affect invasion of ovarian cancer cells and lengthen survival in the SKOV3 xenograft model could be through regulation of the EMT process. Using TGF-β1 treatment to induce EMT in eight ovarian cancer cell lines, we first sought to establish the relationship between TG2 and EMT in these cell lines by assessing the EMT genes *CDH2, SNAI1*, and *VIM*. TGF-β1 stimulated expression of at least one EMT marker in six of the cell lines and increased *TGM2* in five of the cell lines (**Figure 2B**; **Supplementary Figure S2A**). It is notable that two of the cell lines that did not upregulate *TGM2* in response to TGF-β1 also showed no response by the EMT genes, suggesting a good correlation between *TGM2* expression and the EMT process in these model systems. When treated with TG2 inhibitors AA9, NC9, NF20, or VA4 prior to TGF-β1 stimulation, there were no effects on the expression of *TGM2* or the EMT markers *CDH2*, *SNAI1*, or *VIM* in any of the cell lines relative to the DMSO control, indicating that blocking the transamidation catalytic and GTP binding functions of TG2 does not alter EMT gene expression in these cell lines (**Figure 2C**; **Supplementary Figure S2B**). Therefore, inhibition of TG2 may not have prolonged survival in the SKOV3 xenograft models through regulation of the EMT process. Since the survival studies were conducted in NSG mice, the absence of a functional immune system may have masked any significant impact on survival, prompting us to further investigate the role of TG2 within the TME using syngeneic models.

### Lack of TG2 in the TME prolongs survival by delaying metastasis in ID8 *Trp53^-/-^ Brca1^-/-^* tumor-bearing mice

**Figure 3:**
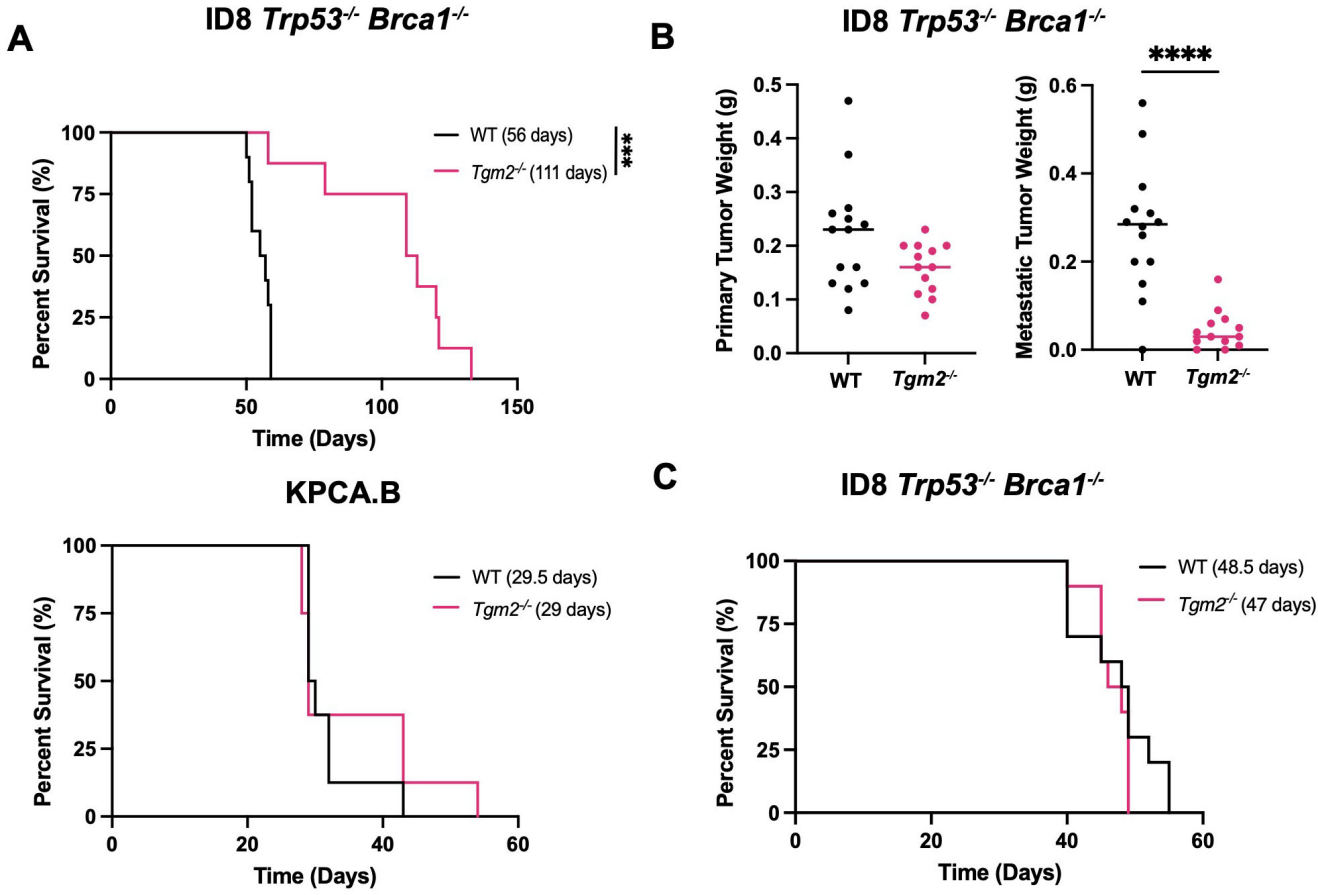
Absence of TG2 in the TME delays disease progression in the ID8 *Trp53^-/-^ Brca1^-/-^* model when cells are injected intrabursally. Wild-type (WT; n=8-10) and *Tgm2* knockout (*Tgm2^-/-^*; n=8) mice receiving intrabursal injections of **(A)** ID8 *Trp53^-/-^ Brca1^-/-^* (top) or KPCA.B murine ovarian cancer cells (bottom) were monitored until humane endpoint to determine differences in survival. Survival is presented in a Kaplan-Meier plot and differences were determined by log-rank test. ***p<0.001. **(B)** WT (n=13) and *Tgm2^-/-^* (n=13) mice received bilateral intrabursal injections of ID8 *Trp53^-/-^ Brca1^-/-^* cells. At 6 weeks after injection, mice were euthanized, and primary tumor and metastases weights were noted. Differences was determined by unpaired Student’s t test. ****p<0.0001. **(C)** WT (n=10) and *Tgm2^-/-^* (n=10) mice receiving intraperitoneal injections of ID8 *Trp53^-/-^ Brca1^-/-^* cells were monitored until humane endpoint to determine differences in survival. Survival is presented in a Kaplan-Meier plot, and significant differences were determined by log-rank test.

To investigate the contribution of TG2 in the TME and potentially correlate it to tumor progression, we used two syngeneic models of ovarian cancer, each with unique metastatic properties and similar TG2 expression (**Supplementary Figure S3**). The ID8 *Trp53^-/-^ Brca1^-/-^* model has mutations commonly found in patients with the high-grade serous subtype of ovarian cancer and is highly metastatic as mice injected with cancer cells orthotopically (intrabursal) succumb to disease due to peritoneal dissemination and ascites accumulation (33, 34). In contrast, in preliminary experiments, we found that KPCA.B cells (*Trp53* ^−/−R172H^ *Ccne1* ^OE^ *Akt2* ^OE^ *KRAS* ^G12V^) are less metastatic and succumb to disease due to the large size of primary orthotopic tumors rather than accumulation of ascites or metastatic disease. To study the role of TG2 in metastasis, we injected ID8 *Trp53^-/-^ Brca1^-/-^* or KPCA.B cells intrabursally into both wild-type and *Tgm2* knockout mice. Survival was significantly prolonged in the ID8 *Trp53^-/-^ Brca1^-/-^* model in the *Tgm2* knockout mice with a median survival of 111 days, compared to the wild-type mice with a median survival of 56 days (**Figure 3A**). No difference in survival was observed in the KPCA.B model (**Figure 3A**). We then analyzed differences in disease progression 6 weeks after intrabursal injections in the ID8 *Trp53^-/-^ Brca1^-/-^* model. While the size of primary tumors was similar between recipient mice, *Tgm2* knockout mice had a significantly lower metastatic burden (by weight) of metastases (**Figure 3B**), and reduced ascites volume and peritoneal cell count (**Supplementary Figure S4**). These results suggest that absence of TG2 in the TME prolonged survival by delaying metastatic dissemination. When ID8 *Trp53^-/-^ Brca1^-/-^* cells were injected intraperitoneally in wild-type and *Tgm2* knockout mice, there was no difference in survival (**Figure 3C**), further emphasizing the importance of TG2 in the TME for dissemination and promoting peritoneal disease. Since it is well established that the immune system plays an important role in ovarian cancer progression (33, 35), we next investigated the changes in the immune composition resulting from absence of TG2 in the TME that might slow the rate of metastasis.

### The absence of TG2 in the TME decreases the abundance of M2 TAMs within ID8 *Trp53^-/-^ Brca1^-/-^* tumors

**Figure 4:**
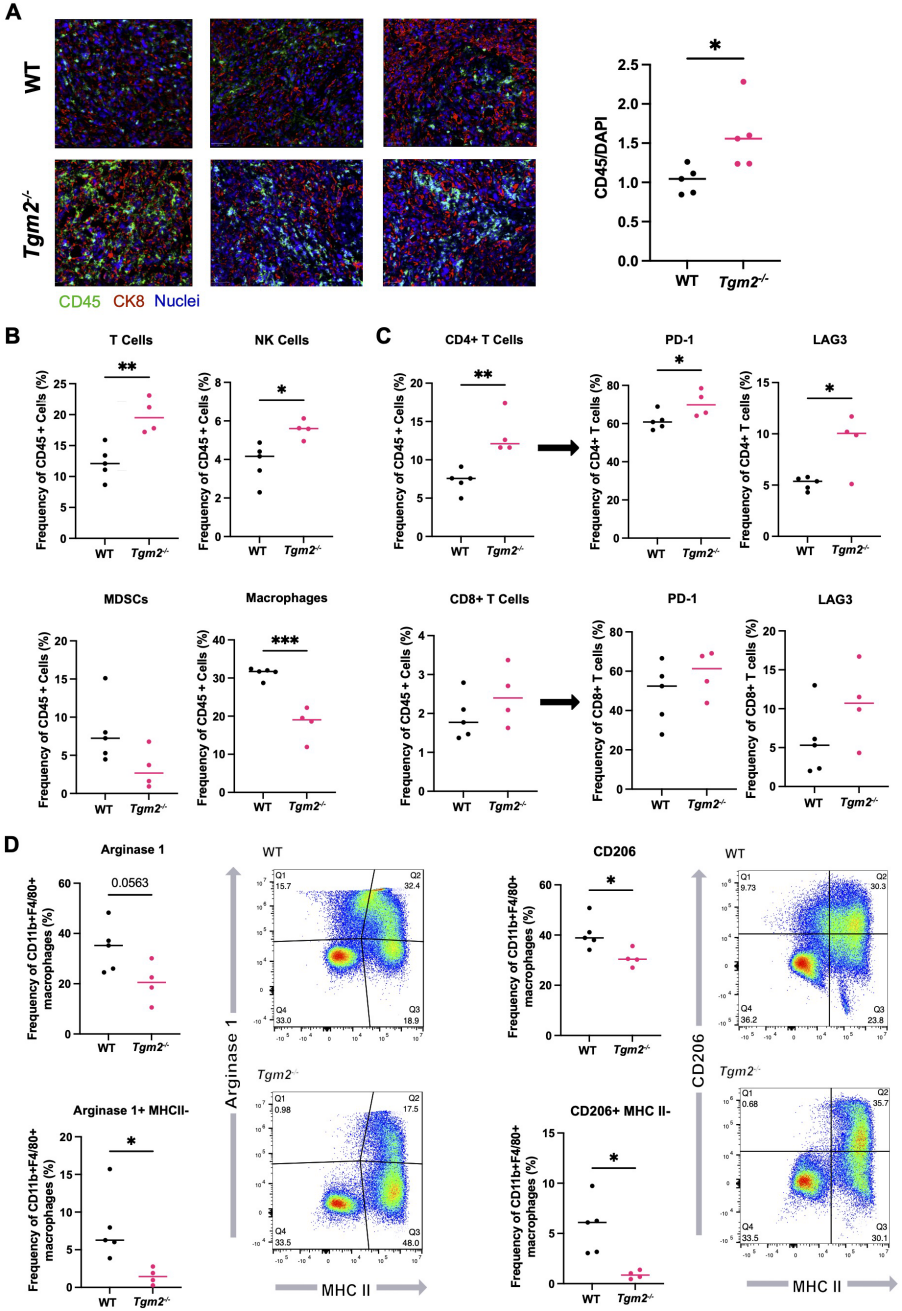
Absence of TG2 in the TME impacts the immune landscape in ID8 *Trp53^-/-^ Brca1^-/-^* orthotopic tumors. Orthotopic tumors from wild-type (WT) and *Tgm2* knockout (*Tgm2^-/-^*) mice injected intrabursally with ID8 *Trp53^-/-^ Brca1^-/-^* ovarian cancer cells were collected 6 weeks after injection. **(A)** CD45 (green) and CK8 (red) staining in tumors to assess immune infiltration (n=5 per experimental group, 3 shown). CD45 was quantified and normalized to DAPI (blue). Significant differences were determined by unpaired Student’s t test. *p<0.05 **(B)** Frequency of specific immune cell types in the orthotopic tumors. **(C)** Frequency of CD4+ and CD8+ T cells and frequency of T cells expressing markers of inhibition/exhaustion PD-1 and LAG3. **(D)** Frequency of macrophages expressing the canonical M2 markers Arginase 1 and CD206, as well as MHC II. Cell frequencies in B-D were assessed by flow cytometry (n=4–5 per experimental group). Significant differences were determined by unpaired Student’s t test. *p<0.05 **p<0.01, ***p<0.01.

Immunosuppression is a dominant process leading to tumor progression in ovarian cancer (5). We therefore compared the immune composition of ID8 *Trp53^-/^* ^-^ *Brca1^-/-^* tumors that developed in the presence or absence of TG2 in the TME to determine if the observed increase in survival could be due to TG2 playing a role in modulating the immune system (**Supplementary Figure S5**). Notably, there was increased immune infiltration in orthotopic tumors isolated from *Tgm2* knockout mice (**Figure 4A**). In the tumors from wild-type mice, the most abundant immune cells were TAMs (**Figure 4B**), which is consistent with previous literature (36). However, the increased immune infiltration in the tumors from *Tgm2* knockout mice was due to an increase in the frequency of T cells and NK cells (**Figure 4B**). Further analysis of the T cells revealed an increase in the proportion of CD4+ T cells, which also had increased expression of PD-1 and LAG3, indicating increased CD4+ T cell activation/exhaustion in the *Tgm2* knockout mice (**Figure 4C**). No differences in frequency or phenotype of CD8+ T cells were noted (**Figure 4C**).

The decreased frequency of TAMs within the *Tgm2* knockout mice was associated with a decrease in M2 TAMs, as reflected by a smaller proportion of Arginase 1- and CD206-expressing TAMs in the tumors (**Figure 4D**). Arginase 1 and CD206 are established markers of M2 TAMs (37). A consistent shift in the TAM phenotype from the M2 immunosuppressive and pro-tumoral phenotype to the M1 inflammatory and anti-tumoral phenotype was also observed. There was a shift from Arginase 1(+) MHC II(-) populations in the tumors from wild-type mice to more MHC II(+) Arginase 1(-) populations in tumors from the *Tgm2* knockout mice indicating overall more anti-tumoral properties of the TAMs present in the tumors from *Tgm2* knockout mice (**Figure 4D**).

### Absence of TG2 in the TME changes the immune profile in the peritoneal cavity

**Figure 5:**
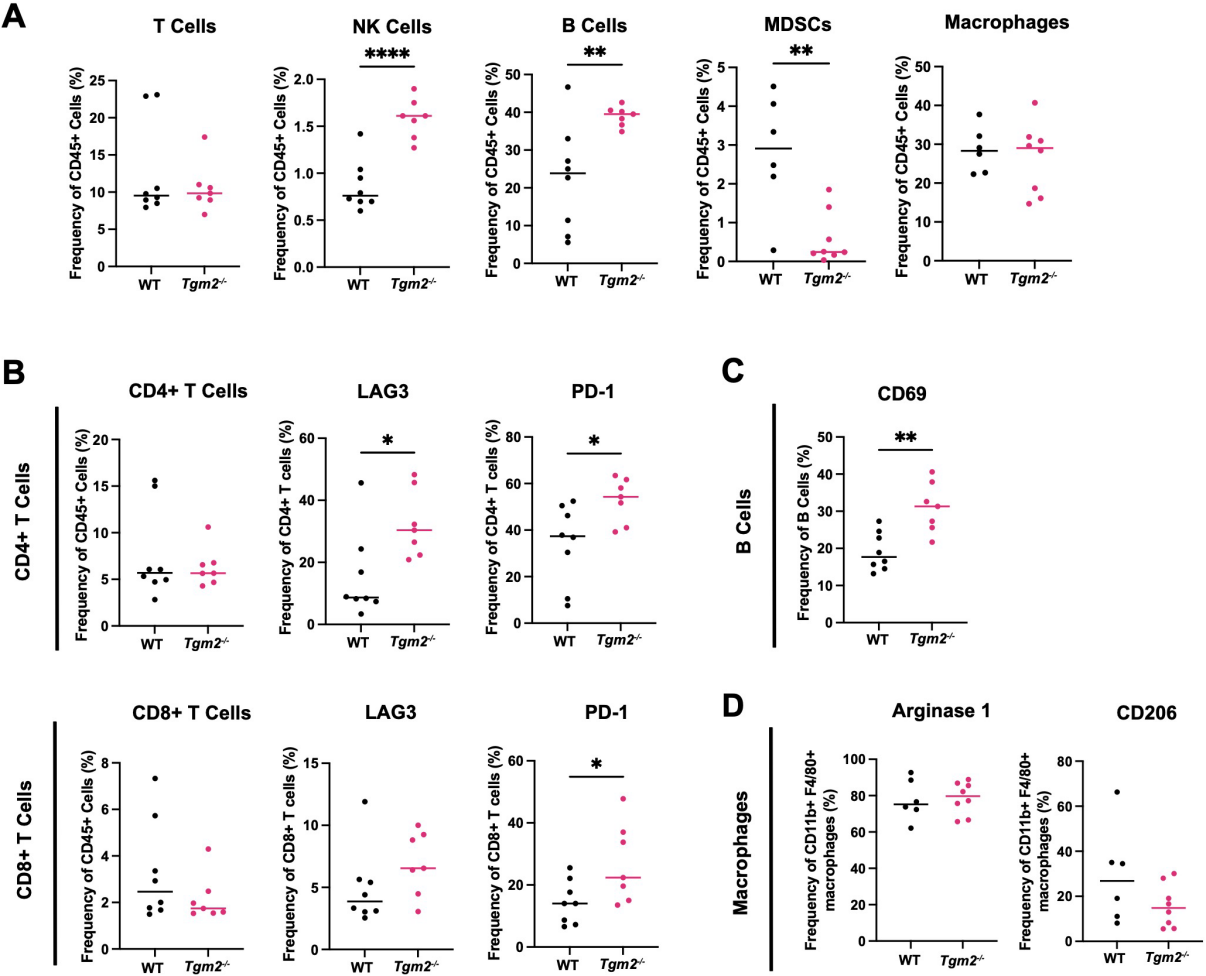
Absence of TG2 in the TME alters peritoneal lymphoid and myeloid progenitor immune cell populations. Cells from peritoneal wash from wild-type (WT) and *Tgm2* knockout (*Tgm2^-/-^*) mice injected intrabursally with ID8 *Trp53^-/-^ Brca1^-/-^* ovarian cancer cells were collected 6 weeks after injection. **(A)** Frequencies of T cells, NK cells, B cells, MDSCs, and macrophages were determined by flow cytometry. Frequency of cells expressing **(B)** activation/inhibition/exhaustion markers LAG3 and PD-1 on CD4+ and CD8+ T cells, **(C)** activation marker CD69 on B cells, and **(D)** M2 markers Arginase 1 and CD206 on macrophages, were assessed by flow cytometry (n=7–8 per experimental group). Significance was determined by unpaired Student’s t test. *p<0.05, **p<0.01, ****p<0.0001.

We analyzed the immune populations present in the ascites within the peritoneal cavity 6 weeks after intrabursal injections of ID8 *Trp53^-/-^ Brca1^-/-^* cells to further understand how TG2 may promote disease progression (**Supplementary Figure S6**). In wild-type mice, the TAMs were the most abundant immune cell type in the peritoneal cavity (**Figure 5A**), similar to the orthotopic tumors. *Tgm2* knockout mice harbored an increased proportion of NK cells and B cells, a decrease in myeloid-derived suppressor cells (MDSCs), and no differences in the frequency of T cells and TAMs compared to wild-type mice (**Figure 5A**). As with orthotopic tumors, there was an increased frequency of CD4+ T cells expressing LAG3 and PD-1 (**Figure 5B**). The peritoneal cells in *Tgm2* knockout mice also showed an increase in frequency of CD8+ T cells expressing PD-1 (**Figure 5B**) and an increased frequency of B cells expressing the activation marker CD69 (**Figure 5C**). No differences were observed in the frequency of TAMs expressing the canonical M2 immunosuppressive markers Arginase 1 and CD206 (**Figure 5D**). The observation that TAMs are the most abundant immune cell in both primary tumors and ascites, combined with the finding that their phenotype differs in primary tumors but not in the peritoneal cavity, led us to further investigate the role of TG2 in macrophage polarization as a potential regulator of the anti-tumor immune response.

### Lack of TG2 or TG2 inhibition does not affect macrophage polarization *in vitro*

**Figure 6:**
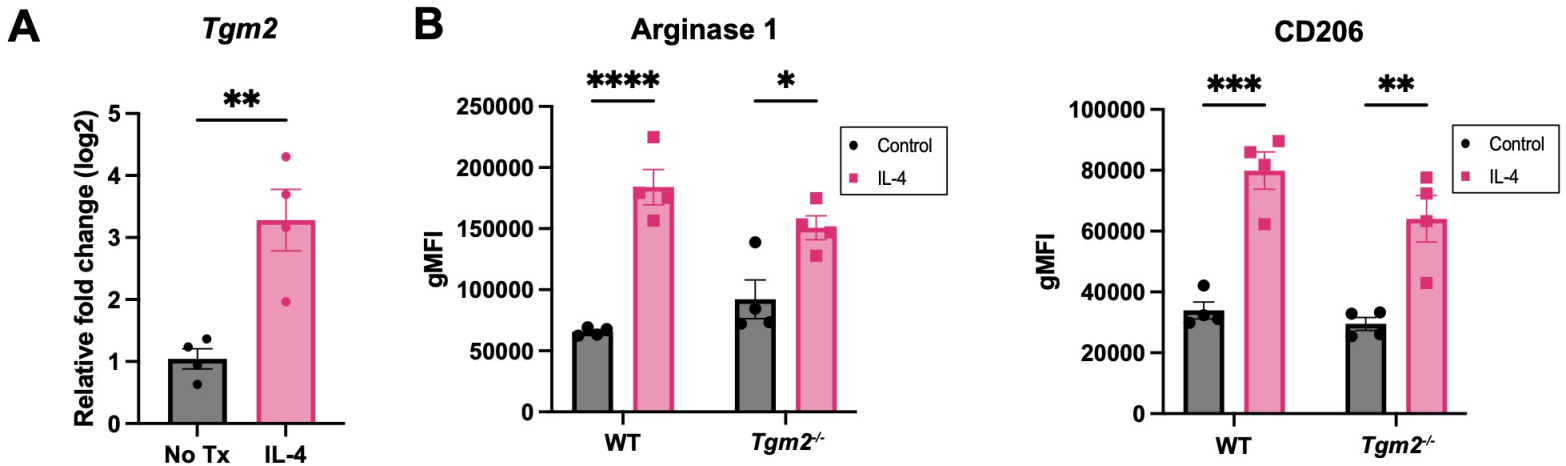
Absence of TG2 in BMDMs does not alter expression of M2 markers induced with IL-4. **(A)** Expression of *Tgm2* is increased in BMDMs when stimulated with IL-4 at 20ng/ml for 48 hours as determined by qPCR. Values shown are the mean ± SEM (n=4 per experimental group). Significant difference was determined by unpaired Student’s t test. **p<0.01. **(B)** Protein expression of polarization markers Arginase 1 and CD206 of WT and *Tgm2^-/-^* BMDMs after IL-4 stimulation was determined by flow cytometry. Histograms show geometric mean fluorescence intensity (gMFI) ± SEM (n=4 per experimental group). Significant differences were determined by two-way ANOVA and Tukey’s *post-hoc* test. *p<0.05, **p<0.01, ***p<0.001, ****p<0.0001.

TG2 has been shown to be upregulated in M2 macrophages (15). Based on the survival and TME experiments described above, we predicted that TG2 might play an important role in promoting immunosuppression in ovarian cancer through modulation of the TAM phenotype. When bone-marrow-derived macrophages (BMDMs) from wild-type mice were polarized to an M2 phenotype by treatment with IL-4, there was an increase in the expression of *Tgm2* (**Figure 6A**), which validates previous findings (38). BMDMs from wild-type and *Tgm2* knockout mice polarized to the same extent as indicated by a similar increase in Arginase 1 and CD206 expression when stimulated with IL-4 (**Figure 6B**). To determine whether TG2 inhibition could prevent or reverse macrophage polarization, the macrophages were treated with the TG2 inhibitors AA9, NC9, VA4, and NCEG2 prior to IL-4 treatment. There was no change in the expression of Arginase 1 or CD206 (**Supplementary Figure S7A**). Similarly, when treated with the same TG2 inhibitors after IL-4 stimulation, no differences in Arginase 1 and CD206 expression were observed (**Supplementary Figure S7B**). Collectively, these findings show that abolishing TG2 activities by gene deletion or inhibition had no significant effect on macrophage polarization *in vitro*.

### B cells play a key role in slowing ovarian tumor development in the absence of TG2

**Figure 7:**
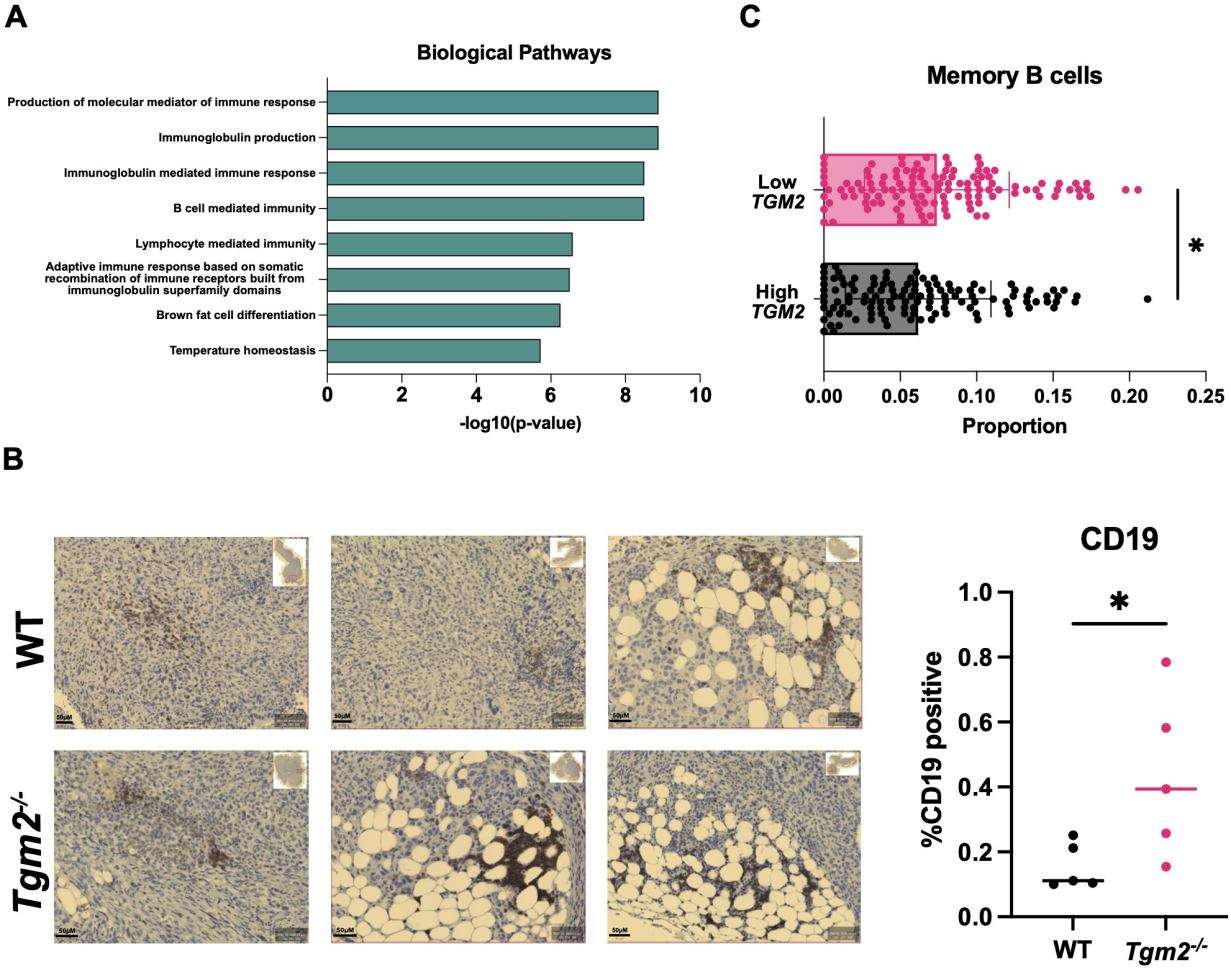
Absence of TG2 in the TME is associated with increased B cell activation and abundance in orthotopic tumors. Primary tumors from wild-type (WT) and *Tgm2* knockout (*Tgm2^-/-^*) mice injected intrabursally with ID8 *Trp53^-/-^ Brca1^-/-^* ovarian cancer cells were collected 6 weeks after injection. **(A)** RNA sequencing analysis shows the increased Gene Ontology biological processes (n=3 per experimental group). **(B)** Immunohistochemical detection of the B cell marker CD19 in orthotopic tumors from *Tgm2^-/-^* and wild-type mice. CD19 was quantified relative to the area of the tumor (n=5 per experimental group). The statistically significant difference was determined by unpaired Student’s t test. *p<0.05 **(C)** CIBERSORT estimated memory B cell immune proportion in low *TGM2* (n=122) and high *TGM2* (n=122) expressing human ovarian tumours from TCGA data. The statistically significant difference was determined by unpaired Student’s t test. *p<0.05.

Since interfering with TG2 expression and activity had no direct effect on macrophage polarization, we performed RNA sequencing (RNA-seq) on the orthotopic ID8 *Trp53^-/-^ Brca1^-/-^* tumors 6 weeks after intrabursal injections to understand the transcriptional programming involved in the delayed disease progression observed in the *Tgm2* knockout mice. Examining GO terms enriched among differentially expressed genes revealed a notable increase in B cell activation, with gene sets including positive regulation of B cell activation, humoral immune response mediated by circulating immunoglobulin, and B cell receptor signaling pathway (**Figure 7A**). Immunohistochemical analysis supported these results showing an increase in B cell frequency within the orthotopic tumors, with a tendency to cluster in adipocyte-rich areas (**Figure 7B**). These findings suggest that changes in the TME immune profile may be due to B cell recruitment and activation. To strengthen our findings, we used the CIBERSORT algorithm to estimate B cell immune proportions in human ovarian tumor samples from The Cancer Genome Atlas (TCGA) with high and low *TGM2* expression. We found that the proportion of memory B cells was increased in the lower-expressing *TGM2* tumors (**Figure 7C**) which is in line with our *in vivo* findings that demonstrate absence of TG2 in the TME is associated with increased B cell recruitment and activation.

In summary, the results from the flow cytometry and RNA-seq analysis of the tumor immune environment suggest that loss of TG2 expression is associated with increases in CD4+ T cells and NK cells, increased B cell activation, and reduction of immunosuppressive TAMs that collectively slow the progression of metastasis in the ID8 *Trp53^-/-^ Brca1^-/-^* model.

## Discussion

Elevated TG2 expression is found in epithelial ovarian tumors and is associated with poor outcomes and increased metastasis (11). This study broadly characterized the role of TG2 in two orthotopic models of ovarian cancer and discovered that TG2 can be a promoter of tumor metastasis and a modifier of the immune microenvironment. We also tested a subset of irreversible TG2 inhibitors for therapeutic efficacy in xenograft models of ovarian cancer.

TG2 is expressed at variable levels in ovarian cancer cell lines. When blocking TG2’s transamidation catalytic and GTP binding functions with novel TG2 inhibitors, there were no notable differences in their proliferation and migration, but there was a significant decrease in invasive capabilities. Although the fibronectin binding ability of TG2 also plays a role in modulating the extracellular matrix, we found that inhibiting the transamidation catalytic and GTP binding functions of TG2 was sufficient to decrease invasive capabilities. In agreement with previous studies (39), we found that TG2 was upregulated during TGF-β1-induced EMT in several ovarian cancer cell lines. However, blocking the transamidation and GTP binding functions of TG2 activity did not affect the induction of canonical EMT genes, suggesting that these inhibitors may be decreasing invasion through an alternative biological process. For example, a study in lung cancer found that increased extracellular TG2 is associated with increased Rac GTPase activity (40). Increased Rac signaling can result in actin remodeling and cell adhesion therefore facilitating invasion of cancer cells independently of the EMT process (40). In ovarian cancer, increased activation of Rac1 was also associated with increased invasion (41). It is therefore possible that blocking both activities of TG2 in our *in vitro* models regulate invasion through different mechanisms. In other cancer types, NC9 has been reported to have notable effects on malignant phenotypes. In epidermal squamous cell carcinoma, NC9 reduced cell survival, EMT, and invasion (42). In mesothelioma, NC9 reduced tumor growth and in breast cancer, NC9 decreased migration but had no impact on invasion (24, 43). In line with our findings, inhibition of the transamidation catalytic and GTP binding functions of TG2 seems to have variable effects depending on the cancer cell type or even the specific cell line.

When evaluating the therapeutic potential of these inhibitors in xenograft models, VA4 delayed disease progression in the SKOV3 model, whereas there were no effects on the OV1946 model. These results suggest that TG2 plays a more significant role in promoting disease progression through GTP binding and/or transamidation activities in SKOV3 cells compared to OV1946 cells. Alternatively, since OV1946 cells express TG2 at higher levels, the dose of inhibitor being used might not have been sufficient to impact survival. Unfortunately, the limited inhibitor solubility did not permit dosing at higher concentrations. Since the SKOV3 xenograft model showed only a minimal increase in survival, we speculated that TG2 may have a more significant impact on disease progression through its activities within the TME. This prompted us to further investigate TG2’s role in shaping the TME with the use of syngeneic mouse models.

Recent studies have shed light on the importance of the TME in promoting disease progression particularly through immunosuppression (33, 35). There are limited studies investigating the potential importance of TG2 in the ovarian TME, but one recent study has shown that TG2 could play a role in modulating the immune response (17). Given this potential, we further investigated the role of TG2 in the TME in syngeneic models and determined if using mice that lacked TG2 expression could confer a therapeutic benefit. We used two syngeneic models, in which cells injected intrabursally allowed us to recreate the process of metastasis. We found that absence of TG2 in the TME did not prolong survival in the less metastatic model (KPCA.B) but did prolong survival in the highly metastatic model, ID8 *Trp53^-/-^ Brca1^-/-^*, as was evident by the *Tgm2* knockout mice having significantly less metastatic disease at 6 weeks after cell injection. Furthermore, when ID8 *Trp53^-/-^ Brca1^-/-^* cells were injected intraperitoneally to eliminate the process of dissemination from a primary tumor, there was no difference in survival between the wild-type and *Tgm2* knockout mice. These findings indicate that TG2 in the TME plays an important role in the metastatic process.

When comparing the immune response in wild-type and *Tgm2* knockout mice, many notable differences were observed. Increased frequency of T cells and NK cells in the orthotopic tumor in the *Tgm2* knockout mice supported an increase in immune activation, especially with an increase in both the frequency of CD4+ T cells and their elevated expression of the exhaustion markers PD-1 and LAG3. In line with the increased survival of these mice, the increase in expression of exhaustion markers could be a result of sustained T cell activation. In complement to this, there was a notable decrease in frequency of TAMs in the *Tgm2* knockout mice, with a decreased M2 immunosuppressive phenotype and a shift towards a more M1 phenotype. Overall, the absence of TG2 in the TME promoted a more anti-tumoral immune response in the orthotopic tumors.

The ascites and peritoneal cavity also had notable differences in the immune microenvironment in the absence of TG2. An increase in NK cell and B cell frequencies was observed, but no differences in the frequencies of T cells and TAMs. Even without a change in T cell abundance, there was still an increase in the frequency of CD4+ T cells expressing LAG3 and PD-1, which could indicate sustained T cell activation leading to exhaustion. Increased frequency of activated B cells expressing CD69 was also evident in the *Tgm2* knockout mice and increased B cell activation has been associated with an anti-tumoral response and improved overall survival in several cancer types including breast, prostate, lung, pancreatic, and ovarian cancer (44). Interestingly, there were no differences in the polarization phenotype of TAMs between the *Tgm2* knockout and wild-type mice. When further investigating the role of TG2 in macrophage polarization *in vitro*, no differences were observed in the extent of polarization when comparing wild-type BMDMs to *Tgm2* knockout BMDMs and when irreversibly inhibiting TG2’s transamidation catalytic and GTP binding functions. This indicates that the polarization status of the macrophages is affected by an alternative mechanism by which TG2 acts on the immune system. Understanding the TME is challenging due to the complex interactions between immune cells, stromal cells, and cancer cells. Previous studies have shown that increased expression of TG2 on macrophages can play an important role in cell adhesion and extravasation leading to increased recruitment of immunosuppressive TAMs (14). These TAMs can also communicate directly or indirectly with other immune cells such as T cells leading to a more immunosuppressive phenotype (45). Collectively, these effects contribute to increased disease progression through an overall increase in an immunosuppressive TME (14, 45). However, in our *in vitro* model system, these factors and interactions are eliminated and thus not a biological representation of processes occurring *in vivo*. Therefore, we sought to explore the various biological processes occurring in the tumors when these important interactions are present through RNA sequencing.

When further analyzing the primary tumors by RNA sequencing, the main biological pathways with increased activation in the *Tgm2* knockout mice involved B cell activation. We also found that human ovarian tumors with low *TGM2* expression had an increased proportion of memory B cells. This is an interesting finding, as recent studies have identified B cells, especially memory B cells, to potentially play an important role in ovarian cancer (3, 46). More specifically, in human high-grade serous ovarian cancer, B cells produce antibodies that target tumor antigens, therefore making cancer cells more recognizable to the immune system (3). They also secrete cytokines and chemokines that contribute to conferring an anti-tumoral response through activation and recruitment of immune cells including NK cells, T cells, and dendritic cells (3). In the ID8 syngeneic ovarian cancer model, B cell depletion resulted in enhanced tumor progression, further exemplifying the importance of B cells in ovarian cancer (3). Collectively, it seems reasonable to speculate that B cells contribute in a similar way to the prolonged survival of the *Tgm2* knockout mice.

This study has further contributed to understanding the role of TG2 in both cancer cells and the TME. We found that TG2 plays an important role in the TME and contributes to the metastatic process, primarily through the regulation of B cell abundance and activity. With new studies shining light on the importance of B cells in ovarian cancer, further investigating the role of TG2 in B cell functions will lead to new therapeutic opportunities.

## Methods

### Cell lines

#### Human cell lines

Human ovarian cancer cell lines PEO1, PEO4, OVCAR8, OV90, and SKOV3 cells were cultured in RPMI media (ThermoFisher; 11875176) with 7.5% fetal bovine serum (FBS, Corning; 35-077-CV). TOV3041G and OV1946 cells were cultured in OSE media (Wisent; 316-030-CL) with 10% FBS. OVCA420 cells were cultured in DMEM (Corning; 10-013-CV) with 10% FBS.

#### Mouse cell lines

The ID8 *Trp53^-/-^ Brca1^-/-^* murine ovarian cancer cell line was generated via CRISPR-Cas9 knockout of *Trp53* and *Brca1* in ID8 cells and provided by Dr. Iain McNeish (34). The cells were cultured in DMEM supplemented with 4% FBS and 0.01mg/mL Insulin-Transferrin-Selenium Supplement (ITSS) (Roche; I1884). The KPCA.B murine ovarian cancer cell line (*Trp53* ^−/−R172H^ *Ccne1* ^OE^ *Akt2* ^OE^ *KRAS* ^G12V^) was generated and provided by Dr. Robert Weinberg and cultured in DMEM (Corning; 10-013-CV) supplemented with 4% FBS, 0.01mg/mL ITSS, and 10 μg/ml of epidermal growth factor (Cedarlane; 2028-EG-200) (47). Cells were maintained at 37°C with 5% CO_2_. Cells were routinely tested for mycoplasma and tested prior to each *in vivo* experiment.

### TG2 inhibitors

Irreversible TG2 inhibitors AA9, NC9, NF20, JA38, VA4, and NCEG2 were prepared as described previously (19, 28, 29). Inhibitors were re-suspended in DMSO for downstream *in vitro* or *in vivo* applications. DMSO was used as a vehicle control for all experiments involving TG2 inhibitors. The selectivity of these inhibitors for TG2 has previously been established using colorimetric transamidase activity assays and GTP binding assays (19, 28, 29).

### Mice

All *in vivo* studies were conducted following protocols approved by the University of Ottawa Animal Care Committee and in accordance with the guidelines of the Canadian Council on Animal Care. C57BL/6 mice were originally obtained from The Jackson Laboratory (#000664) and bred in-house. *Tgm2* knockout mice were generated by Dr. Robert Graham (Victor Chang Cardiac Research Institute, New South Wales, Australia), generously obtained from Dr. Daniela Matei (Northwestern University Feinberg School of Medicine, Chicago, United States), and bred in-house. These models were used for all experiments using the ID8 *Trp53^-/-^ Brca1^-/-^* and KPCA.B cell lines. All mice used for experimentation were between the ages of 6–10 weeks. NOD *scid* gamma (NSG) mice were obtained from The Jackson Laboratory (#005557). These mice were used for all *in vivo* experiments using human cell lines.

### *In vivo* survival studies

#### Xenograft models

NSG mice received intraperitoneal injections of 5 million SKOV3 or OV1946 cells. At 25% of the estimated length of survival (based on previous experiments), mice were treated with TG2 inhibitors NC9 (20mg/kg), VA4 (20mg/kg), or DMSO (%v/v) as a vehicle control dissolved in 20% Captisol three times a week. Mice were continuously monitored for abdominal distension, discomfort, pallor, or pain and euthanized at a humane endpoint.

#### Syngeneic models

C57BL/6 WT and *Tgm2* knockout mice received orthotopic bilateral intrabursal injections of 2μL of PBS containing 1.5e5 ID8 *Trp53^-/-^ Brca1^-/-^* or KPCA.B cells as previously described (48). Mice were monitored for humane endpoint to determine overall survival. In a separate experiment, C57BL/6 WT and *Tgm2* knockout mice received intraperitoneal injections of 5 million ID8 *Trp53^-/-^ Brca1^-/-^* cells. Mice were continuously monitored to assess for abdominal distension, discomfort, pallor, and pain and euthanized at a humane endpoint to determine survival.

### Quantitative PCR

RNA extraction was performed using the RNeasy Mini Kit (Qiagen; 74106) following the manufacturer’s protocol. cDNA was synthesized from RNA using the iScript reverse Transcription Supermix (Bio-Rad; 1708841) or LunaScript RT Supermix (New England BioLabs; M3010X). For quantitative RT-PCR, 2μL of cDNA was added to their respective wells in a 96-well qPCR plate. A total of 8μL of master mix containing 5μL of SsoAdvanced Universal SYBR Green Supermix (Bio-Rad; 1725274), 2.5μL of nuclease free H_2_O, and 0.25μL of reverse and forward primers (10 µM) was added to each well (**Supplementary Table 1**). Primers were optimized to ensure proper efficiency and amplification before use. The 96-well plates were run through the 7500 Fast Real-Time PCR system to determine the level of gene expression for each target gene. Raw data were analyzed using the delta Outdanced CT method to represent relative gene expression.

### Western blot

Cells were lysed with RIPA lysis buffer containing 1X protease inhibitor (Sigma; P8340). Equal amounts of protein were prepared in PBS and SDS sample buffer. Samples were denatured at 100°C for 5 minutes and loaded into wells of an 8-12% polyacrylamide protein gel. Samples were run in 1X SDS running buffer for 45 min at 45mA. Proteins were transferred to a PVDF transfer membrane (ThermoFisher; P188520) in transfer buffer-containing 20% methanol at 100 volts for 90 min. The PVDF transfer membrane was incubated in primary antibody diluted in bovine serum albumin (BSA, BioShop; ALB001) at room temperature overnight at 4°C (**Supplementary Table 2**). The PVDF membrane was then incubated in HRP secondary antibody diluted in BSA for 1 hour at room temperature (**Supplementary Table 2**). The PVDF membrane was washed, incubated in western ECL substrate (Bio-Rad; 1705061), and imaged using the BioRad ChemiDoc imaging system.

### Proliferation assay

Human ovarian cancer cell lines were plated in IncuCyte Image Lock 96-well dishes. Cells were either left untreated, treated with 20 μM of TG2 inhibitors (AA9, NC9, NF20, and VA4), or treated with vehicle control with equivalent v/v% DMSO (0.05%). A concentration of 20 μM was used to limit cell toxicity. The 96-well plates were placed in an IncuCyte machine and phase contrast images were acquired for 72 hours using the IncuCyte Zoom System to determine cell confluence with the goal of assessing proliferation.

### Scratch wound migration

Human ovarian cancer cell lines were plated in IncuCyte Image Lock 96-well dishes and allowed to adhere for 24 hours at 37°C. Scratch wounds were formed in each well using an IncuCyte WoundMaker. Cells were either left untreated, treated with 20 μM of TG2 inhibitors (AA9, NC9, NF20, and VA4), or treated with vehicle control with equivalent v/v% DMSO (0.05%). The 96-well plates were placed in an IncuCyte machine and phase contrast images were acquired for up to 48 hours using the IncuCyte Zoom System to determine rate of wound closure.

### Transwell invasion assay

Human ovarian cancer cell lines were added to serum-free medium, serum-free medium with 20 μM of TG2 inhibitors (AA9, NC9, NF20, and VA4), or serum-free medium with v/v% DMSO (0.05%, vehicle control). Cells were added to the top chamber of Transwell inserts (Corning; 354578) coated with growth factor-reduced Cultrex (R&D Systems; 3433-005-01) in 24-well plates. Complete medium with the respective 20 μM of inhibitor was placed at the bottom of each well. The 24-well plates were incubated at 37°C for 72 hours. Cells that had invaded through the Transwell inserts were fixed with 70% methanol, stained with toluidine blue, and rinsed with double distilled water. Invaded cells were imaged using a Biotek Gen 5 plate reader and counted manually using FIJI software.

### TGF-β1 and TGF-β1 with TG2 inhibitor treatment

Human ovarian cancer cells were seeded and allowed to adhere overnight at 37°C, then treated with 10 ng/mL TGF-β1 (Cedarlane; 240-B-010) for 48 hours. Cells were collected for RNA extraction and quantitative RT-PCR to measure expression of target genes (*TGM2, CDH2, SNAI1, VIM*). Target gene expression was normalized to housekeeping genes *PPIA* and *GUSB*. In a separate experiment, human ovarian cancer cell lines were either left untreated, treated with 20 μM of TG2 inhibitors (AA9, NC9, NF20, and VA4), or treated with vehicle control with equivalent v/v% DMSO (0.05%) for 30 minutes at room temperature. Cells were then treated with 10 ng/mL TGF-β1 for 48 hours before RNA extraction and quantitative RT-PCR to assess expression of target genes (*TGM2, CDH2, SNAI1, VIM*). Target gene expression was normalized to housekeeping genes *PPIA* and *GUSB*.

### Tissue processing for tumor microenvironment analysis

Primary tumors and peritoneal cells were collected from tumor-bearing mice 6 weeks after intrabursal injections of murine ovarian cancer cells (ID8 *Trp53^-/-^ Brca1^-/-^*). Primary tumors were enzymatically digested in C Tubes (Miltenyi; 130-093-237) using the digestion solution from the tumor dissociation kits (Miltenyi; 130-096-730). Red blood cells were lysed with Ammonium-Chloride-Potassium (ACK; Quality Biological; 118-156-101). Cells were counted and washed once more prior to being stained for flow cytometry. For collection of peritoneal cells, 5 ml of PBS + 2 mM EDTA were injected into the peritoneal cavity of each mouse. The abdomen was massaged for 5 minutes, and the peritoneal cavity was opened to collect the fluid. Cells were strained through a filter into a 50 ml falcon tube to ensure a single cell suspension. Red blood cells were lysed with ACK. Remaining cells were washed with PBS + 2% FBS and then stained for flow cytometry.

### Flow cytometry

Single cell suspensions were stained with either Fixable Viability Stain 510 (BD Biosciences; 564406) or Zombie NIR Fixable Viability Kit (Biolegend; 423106). Cells were treated with Fc block (BD Biosciences; 553142) for 5 minutes and then incubated with the antibody master mix (PBS + 2% FBS) for extracellular staining for 20 minutes (**Supplementary Table 3**). Brilliant stain buffer (BD Biosciences; 563794) was added to the master mix if the panel contained a brilliant violet antibody. For intracellular staining, cells were fixed and permeabilized using the Foxp3/Transcription Factor Staining Buffer Set (eBioscience; 501128857) following the manufacturer’s recommendations. The intracellular antibody master mix containing antibodies, permeabilization buffer, and brilliant buffer (if there was a brilliant violet antibody in the panel) were added to each sample and incubated for 40 minutes (**Supplementary Table 3**). Cells were spun down and fixed with 1% paraformaldehyde (PFA). Cells were either run on the Cytek Aurora or the BD Fortessa with UltraComp eBeads (Fisher Scientific; 01-2222-42) used for compensation. Data collected included fluorescent intensity of each fluorophore, percent positive cells and mean fluorescence intensity as analyzed with FlowJo.

### Bone marrow derived macrophages

Bone marrow was isolated from the femur and tibia of wild-type and *Tgm2* knockout mice using PBS + 2% FBS and a 27-gauge needle. Red blood cells were lysed using ACK lysis buffer for 3 minutes. Cells were washed, spun down, and resuspended in DMEM + 10% FBS + 1X penicillin/streptomycin (ThermoFisher; 15140122). Cells were seeded in 6-well plates (350,000/well) and were treated with 20 ng/ml of macrophage colony stimulating factor (Peprotech; AF31502) every 3 days for a total of 7 days to differentiate monocytes into macrophages. Media was changed on day 6 and macrophages were used in polarization experiments.

### BMDM polarization

To polarize BMDMs to an M2 phenotype, BMDMs were treated with IL-4 (Peprotech; AF21414; 20 ng/ml) on day 6 of differentiation for 48 hours and subjected to further downstream analyses. Cells were treated with TG2 inhibitors AA9, NC9, VA4, NCEG2 or DMSO as a vehicle control either 30 minutes prior to IL-4 stimulation or 24 hours after IL-4 stimulation. Macrophage polarization status was determined using flow cytometry to detect protein expression of polarization markers Arginase 1 and CD206. Expression of *Tgm2* was assessed by quantitative RT-PCR.

### Immunohistochemistry

Paraffin blocks containing primary tumors were microtome sectioned at 5 μm. Sections were rehydrated and deparaffinized by immersing slides in xylene, 100% and 95% ethanol, and deionized water. Slides were incubated in pressurized citrate buffer concentrate for antigen unmasking. Once the slides were cooled, the tissues were placed in 3% hydrogen peroxide solution for 5 minutes. Tissues were washed with PBS and blocked using the DAKO protein block solution (DAKO; X090930-2) for 20 minutes. Tissues were incubated in primary antibodies diluted in antibody diluent (DAKO; S302283-2) for 1 hour at room temperature, or overnight at 4°C. Slides were washed with PBS 3 times for 2 minutes each. Secondary antibody diluted in antibody diluent (DAKO; S302283-2) was added to the tissues for 1 hour at room temperature and washed with PBS (**Supplementary Table 4**). Tissues were placed in a solution containing DAB (Sigma; D5637-5G) for 5 minutes. Once rinsed, the tissues were dipped in hematoxylin, ammonia water, and washed with water. Sections were dehydrated in 95% ethanol, 100% ethanol, and xylene. Coverslips were mounted onto slides using PermaFluor Aqueous Mounting Medium (Fisher Scientific; TA-030-FM). Tissues were imaged using the Zeiss AxioScan Z1. Protein abundance was assessed using Orbit analyzer by determining the percentage of positive pixels.

### Immunofluorescence

Primary tumors embedded in frozen OCT blocks were cryostat sectioned at 7 μm. Tissue sections were fixed using 2% PFA at room temperature for 20 minutes. Slides were washed with PBS and then blocked in 10% goat serum (Sigma; G9023) and 0.2% Triton X-100 in PBS for 1 hour. Primary antibodies (**Supplementary Table 4**) were diluted to the appropriate dilution in 10% goat serum and 0.2% Triton X-100 in PBS and left on the tissue to incubate for 1 hour at room temperature or overnight at 4°C and then washed with PBS. Fluorophore-conjugated secondary antibodies were diluted to the appropriate dilution in 10% goat serum in PBS. A 1:300 concentration was used for Hoechst stain (Life Technologies; H3570). Tissues were incubated in secondary antibodies for 1 hour at room temperature and washed with PBS. Coverslips were mounted onto slides using PermaFluor Aqueous Mounting Medium (Fisher Scientific; TA-030-FM), and slides were imaged using a Zeiss Axio Imager M2.

### RNA sequencing of primary orthotopic tumors

Primary tumors were collected from tumor-bearing mice 6 weeks after intrabursal injections of murine ovarian cancer cells (ID8 *Trp53^-/-^ Brca1^-/-^*). RNA extraction of primary tumors was performed using the RNeasy Mini Kit (Qiagen; 74106) following the manufacturer’s protocol. RNA was sequenced using Illumina NovaSeq PE100 (25M reads). Transcript quantification was performed using the Kallisto package, and gene-to-transcript mappings were retrieved using the biomaRt package from the Ensembl database. The data was processed through DESeq2 package, which included filtering out genes with low expression, followed by normalization using estimateSizeFactors function. Differential expression between tumors from *Tgm2* knockout and wild-type mice was assessed using the DESeq function, with a threshold for significance set at an adjusted p-value (q-value) < 0.05 and a log2 fold change (Log2FC) cutoff of |Log2FC| > 1. The clusterProfiler package was used for Gene Ontology (GO) enrichment analysis, focusing on the Biological Process (BP) ontology of upregulated genes in *Tgm2* knockout tumors.

### TCGA analysis for B cells

RNA-sequencing gene expression data from ovarian tumors were obtained from The Cancer Genome Atlas (49) and analyzed to investigate the relationship between *TGM2* expression and immune cell infiltration. Immune cell proportions were estimated using the CIBERSORT algorithm. Samples with missing *TGM2* expression values were excluded. One outlier with expression values beyond the 99th percentile was removed to reduce skew. Samples were then stratified into “High” and “Low” *TGM2* expression groups based on the median value. The relative proportions of memory B cells were then estimated using the CIBERSORT algorithm.

### Statistical analysis

Statistical analyses were performed using Prism 9.0 (GraphPad Software Inc.). Student’s t-test was used to determine statistical differences between two groups. One-way ANOVA followed by Dunnett’s *post-hoc* test was used to assess differences among three or more groups. Repeated measures two-way ANOVA was used to assess differences between groups influenced by two independent factors, one of which was time. The log-rank (Mantel-Cox) test was used to determine statistical differences in survival analyses. Two-way ANOVA followed by Tukey’s *post-hoc* test was used to evaluate differences between groups with two independent factors.

## Data Availability

The RNA-seq data is available at NCBI GEO Accession GSE303025.
