# Supplementary material for: Transglutaminase 2 regulates ovarian cancer metastasis by modulating the immune microenvironment
Source: Front Immunol. 2025 Jul 24;16:1639853. doi: 10.3389/fimmu.2025.1639853 (PMC12328189; doi:10.3389/fimmu.2025.1639853)
Supplement: Supplementary file 1 [file DataSheet1.docx]

**Supplemental Figure S1: Effect of TG2 inhibitor treatment on proliferation and migration of TOV3041G and SKOV3 ovarian cancer cells.** Human ovarian cancer cell lines TOV3041G and SKOV3 were treated with TG2 inhibitors AA9, NC9, NF20, VA4 or DMSO as a vehicle control. A) Proliferation was assessed based on cell confluency at a TG2 inhibitor dose of 20 μM. Data were normalized to confluence at the 0-h time point. B) Migration was assessed by scratch-wound assay of cells treated with TG2 inhibitors at a dose of 20 μM. Data are represented by relative wound density. For all figures, the values shown are the mean (n=3) and. significant differences were determined by repeated measure two-way ANOVA.

**Supplemental Figure S2: Effects of TGF-β1 and TG2 inhibitor treatment on *TGM2* and EMT gene expression.** A) Cells were treated with TGF-β1 (10 ng/ml) for 48 hours and expression of *TGM2* and the EMT genes *CDH2, SNAI1, and VIM* was assessed by qPCR. Values shown are the mean ± SEM (n=3). Statistical significance was determined by one sample t and Wilcoxon tests. *p≤0.05, p**≤0.01. B) Cells were treated with TG2 inhibitors AA9, NC9, NF20, VA4 (20 μM) or DMSO as a vehicle control for 30 minutes prior to TGF-β1 (10 ng/ml) treatment for 48 hours. Expression of *TGM2* and the EMT genes *CDH2, SNAI1, and VIM* was assessed by qPCR. Values shown are the mean ± SEM (n=3). Significant differences were determined by one-way ANOVA and Dunnett’s post-hoc test.

**
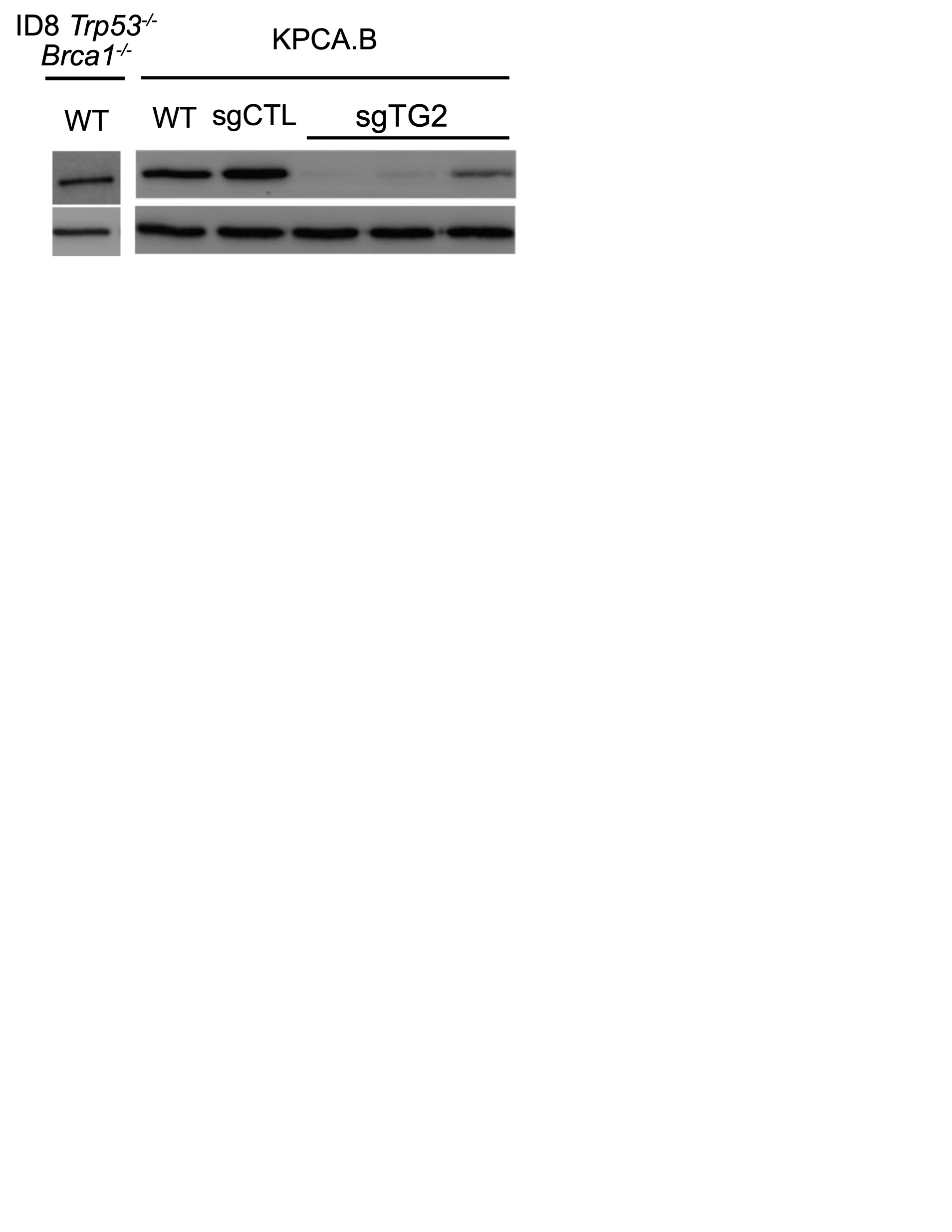
**

**Supplemental Figure S3: TG2 protein expression in ID8 *Trp53^-/-^ Brca1^-/-^* and KPCA.B cell lines.** TG2 expression in ID8 *Trp53^-/-^ Brca1^-/-^* and KPCA.B cells was determined by western blot. Specificity of the TG2 antibody was ensured by evaluating the expression of TG2 in TG2 knockout KPCA cells using 3 guide RNAs (sgTG2) relative to the guide control (sgCTL).

**
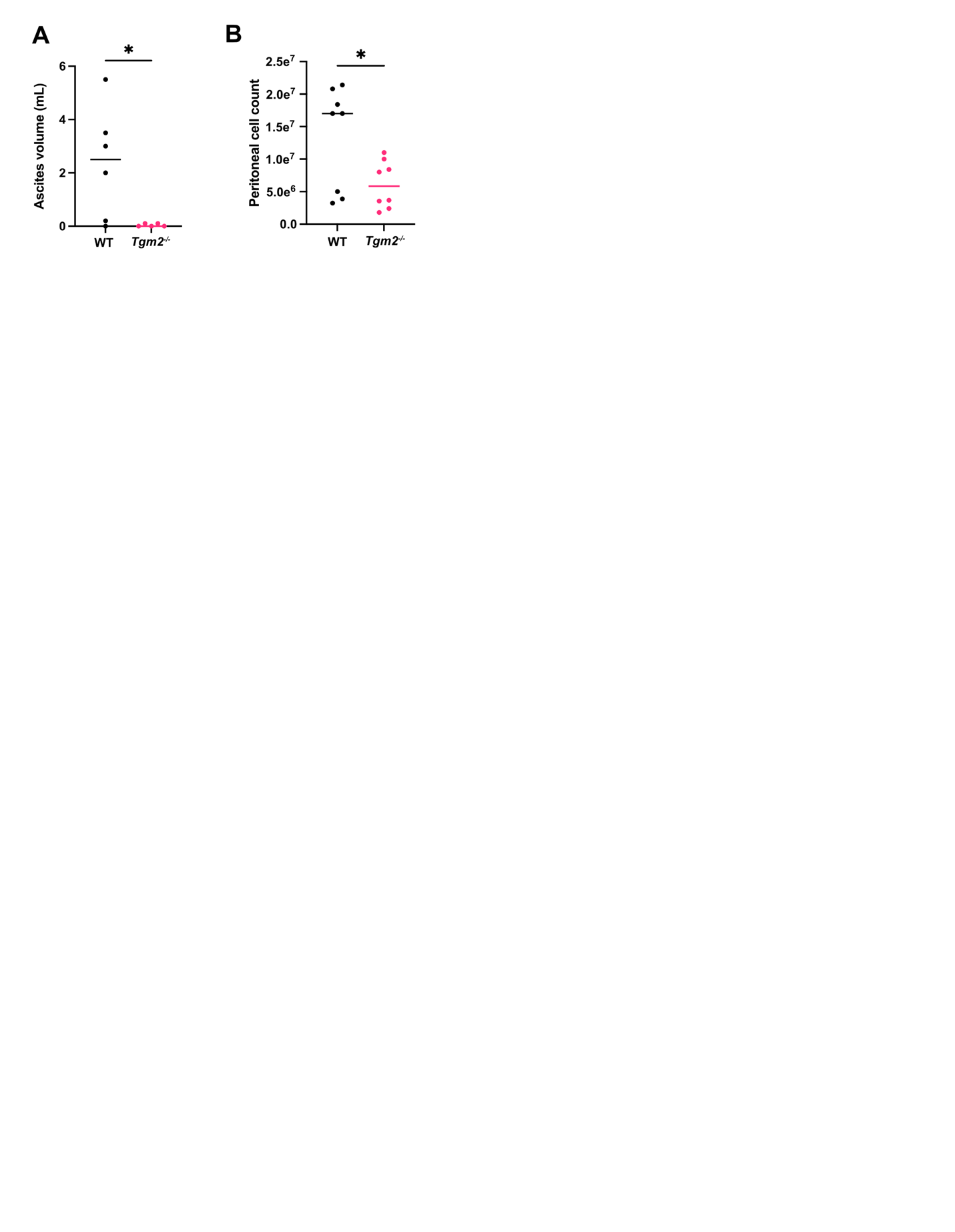
**

**Supplemental Figure S4: Ascites volume and peritoneal cell count in WT and *Tgm2^-/-^* mice 6 weeks after intrabursal injection with ID8 *Trp53^-/-^* *Brca1^-/-^* cells.** Wild-type (WT) and *Tgm2* knockout (*Tgm2^-/-^*) mice received bilateral intrabursal injections of ID8 *Trp53^-/-^ Brca1^-/-^* cells. At 6 weeks after injection, mice were euthanized, and (A) ascites volume (n=5-6) and (B) peritoneal cell count (n=8) were noted. Significant differences were determined by unpaired Student’s t test. *p≤ 0.05.

**
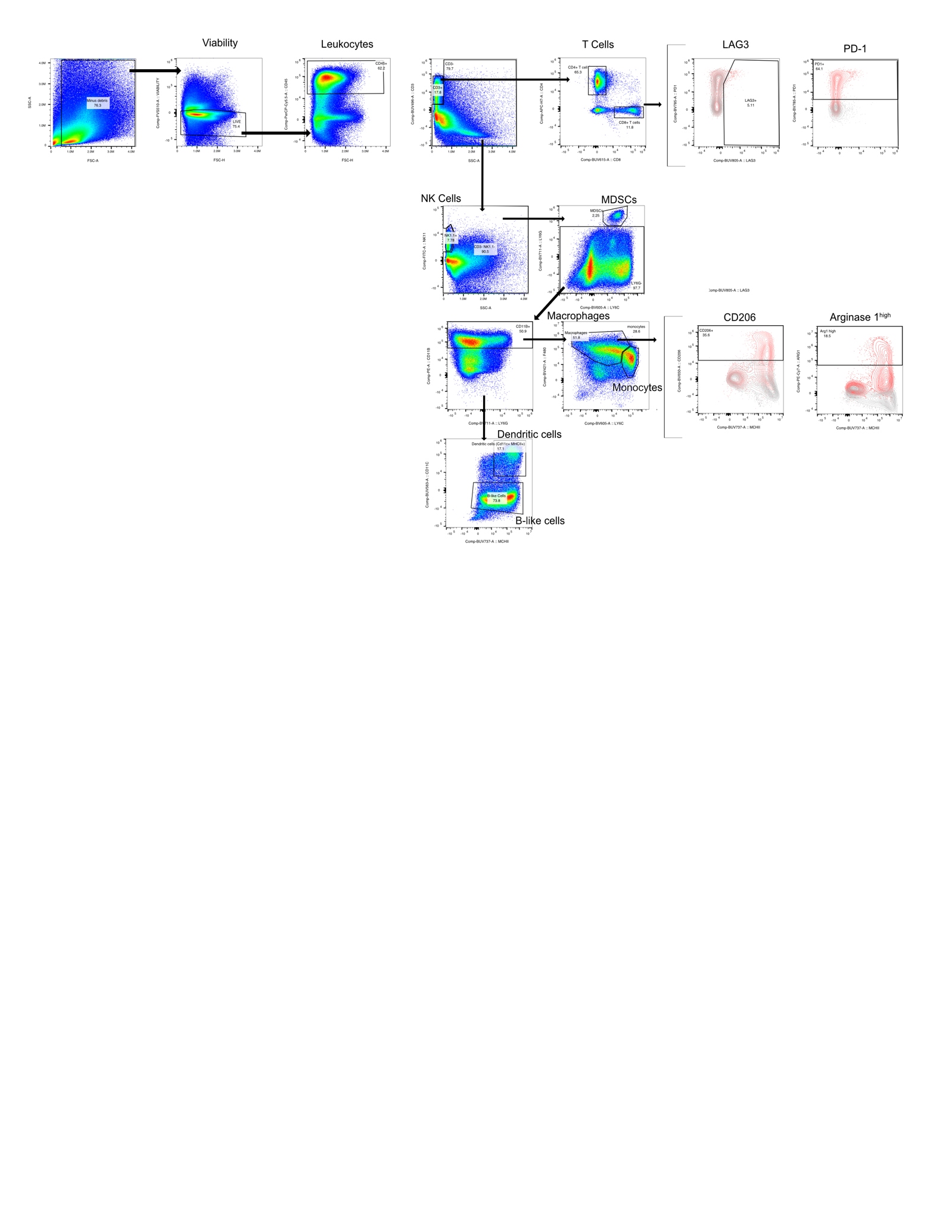
**

**Supplemental Figure S5: Gating strategy for flow cytometry analysis of the primary tumors.** The gating strategy for the immune orthotopic tumors was as follows: Exclusion of cell debris, viability, CD45+ leukocytes, CD3+ T cells, CD3- NK1.1+ NK cells, Ly6C+ Ly6G+ MDSCs, CD11b+ Ly6C+ F4/80+ monocytes, CD11b+ Ly6C- F4/80+ macrophages, and CD11c+ MHCII positive dendritic cells. CD4+ T cells were characterized as CD3+ CD4+ CD8- and CD8+ T cells were characterized as CD3+ CD4- CD8+. LAG3, PD-1, CD206, and Arginase 1 were used to further phenotype immune populations. In these plots, the fluorescence minus one is shown in grey compared to one of the tumor samples in red.

**
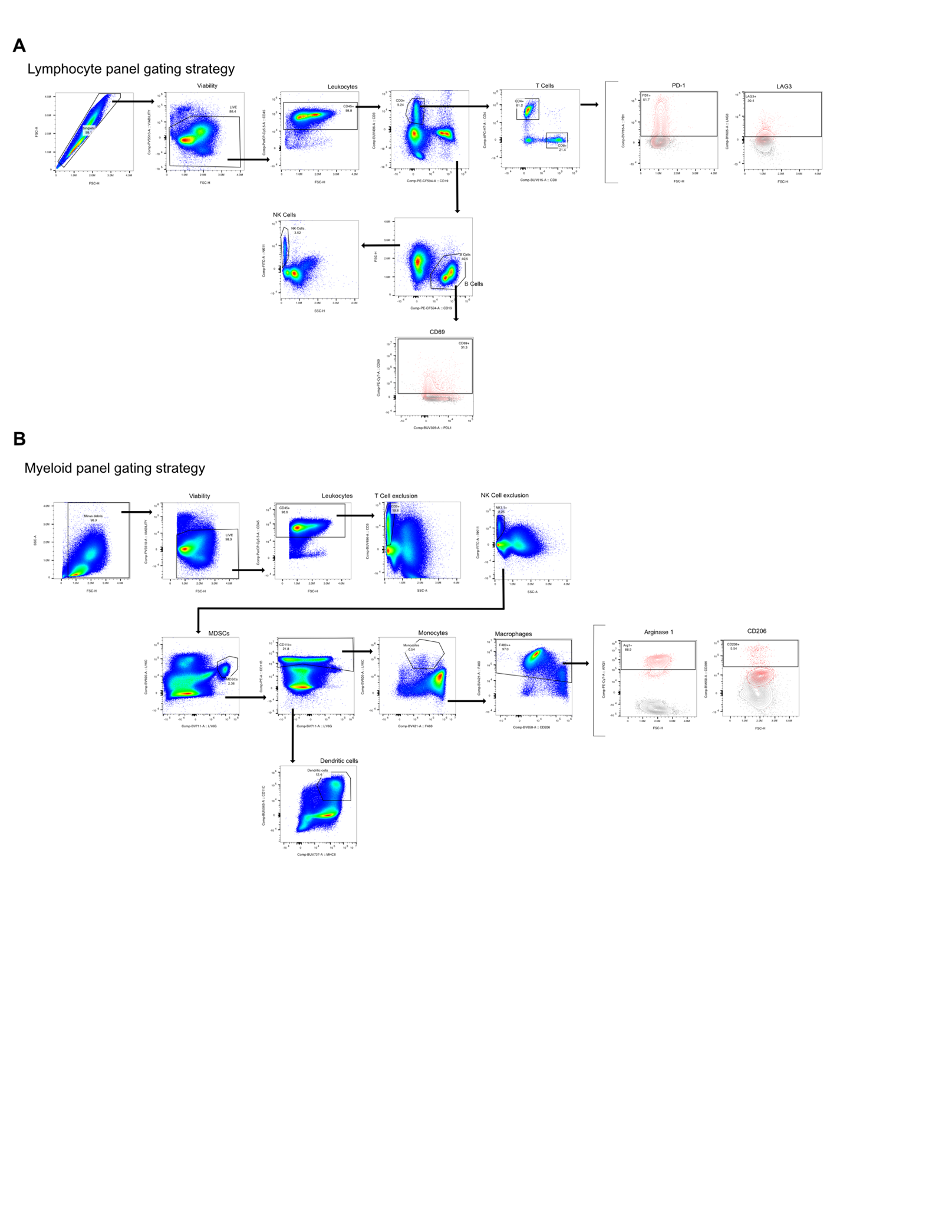
**

**Supplemental Figure S6: Gating strategy for flow cytometry analysis of the peritoneal cells.** A) Gating strategy for the lymphocyte cell population was as follows: Exclusion of cell debris, viability, CD45+ leukocytes, CD3+ T cells, CD19+ B cells, and CD3- NK1.1+ NK cells. CD4+ T cells were characterized as CD3+ CD4+ CD8- and CD8+ T cells were characterized as CD3+ CD4- CD8+. LAG3, PD-1, CD69 were used to further phenotype immune populations. In these plots, the fluorescence minus one is shown in grey compared to one of the tumor samples in red. B) Gating strategy for the myeloid cell population was as follows: Exclusion of cell debris, viability, CD45+ leukocytes, CD3+ T cell exclusion, NK1.1+ NK cell exclusion, Ly6C+ Ly6G+ MDSCs, CD11b+ Ly6C+ F4/80+ monocytes, CD11b+ Ly6C- F4/80+ macrophages, and CD11c+ MHCII positive dendritic cells. CD206, and Arginase 1 were used to further phenotype immune populations. In these plots, the fluorescence minus one is shown in grey compared to one of the tumor samples in red.

**Supplemental Figure S7: TG2 inhibitors AA9, NC9, VA4, and NCEG2 do not prevent or reverse macrophage polarization *in vitro*.** A) BMDMs were pre-treated with the TG2 inhibitors AA9, NC9, VA4, and NCEG2 (20μM) and DMSO as a vehicle control for 30 minutes and then stimulated with IL-4 for 48 hours. B) BMDMs were stimulated with IL-4 for 24 hours and then treated with TG2 inhibitors AA9, NC9, VA4, and NCEG2 (20μM) with DMSO as a vehicle control for an additional 48 hours. Protein expression of M2 markers Arginase 1 and CD206 was assessed using flow cytometry by measuring gMFI ± SEM (n=4). Significance determined by one-way ANOVA and Dunnett’s post-hoc test. *p≤0.05, **p≤0.01, ***p≤ 0.001, ****p≤0.0001

**Supplemental Table 1: qPCR Primers for Human and Mouse Data**

| **Gene** | **Forward primer 5’-3’** | **Reverse primer 5’-3’** |
| --- | --- | --- |
| Hrpt1 (Mouse) | TCAGTCAACGGGGGACATAAA | GGGGCTGTACTGCTTAACCAG |
| Ppia (Mouse) | AGGGTGGTGACTTTACACGC | GATGCCAGGACCTGTATGCT |
| Tgm2 (Mouse) | TGGAACTTTGGGCAGTTCGAG | CATAGATGGGACTGCTGCGG |
| PPIA (Human) | AGACAAGGTCCCAAAGAC | ACCACCCTGACACATAAA |
| GUSB (Human) | AGCCAGTTCCTCATCAATGG | GGTAGTGGCTGGTACGGAAA |
| YWHAZ (Human) | GGTGATGACAAGAAAGGGATTG | GCGTGCTGTCTTTGTATGACTC |
| TGM2 (Human) | AGGGTGACAAGAGCGAGA TG | TGGTCA TCCACGACTCCAC |
| CDH2 (Human) | ACTCAACCCAAACAGGTGCC | AGCGTGTTGCTCTTGTCCTT |
| VIM (Human) | CGGGAGAAA TTGCAGGAGGA | AAGGTCAAGACGTGCCAGAG |
| SNAI1 (Human) | GGTTCTTCTGCGCTACTGCT | TGCTGGAAGGTAAACTCTGGAT |

**Supplemental Table 2: Western Blot Antibodies**

| **Antibody** | **Company** | **Catalogue number** | **Dilution** | **Incubation** |
| --- | --- | --- | --- | --- |
| TG2 | Cell Signaling | 56184 | 1:1000 | 4°C overnight |
| Lamin A/C | Cell Signaling | 4777 | 1:1000 | 4°C overnight |
| Goat Anti-Rabbit IgG (H+L) - HRP | Bio-Rad | 1706515 | 1:5000 | RT 1 hr |
| Goat Anti-Mouse IgG (H+L) – HRP | Bio-Rad | 1706516 | 1:5000 | RT 1 hr |

**Supplemental Table 3: Flow Cytometry Antibodies**

| **Antibody** | **Fluorochrome** | **Company** | **Catalogue Number** | **Dilution** |  |
| --- | --- | --- | --- | --- | --- |
| CD45 | PerCP-Cy5.5 | BD Biosciences | 550994 | 1:200 |  |
| CD3e | BUV496 | BD Biosciences | 612955 | 1:200 |  |
| CD4 | APC-H7 | BD Biosciences | 560181 | 1:200 |  |
| CD8 | BUV615 | BD Biosciences | 613004 | 1:200 |  |
| CD11b | PE | BD Biosciences | 557397 | 1:800 |  |
| CD11c | BUV563 | BD Biosciences | 749040 | 1:200 |  |
| CD19 | PE-CF594 | BD Biosciences | 562291 | 1:400 |  |
| CD206 | BV650 | Biolegend | 141723 | 1:200 |  |
| CD69 | PE-Cy7 | BD Biosciences | 552879 | 1:200 |  |
| F4/80 | BV421 | BD Biosciences | 565411 | 1:150 |  |
| Arginase 1 | PE-Cy7 | Invitrogen | 25-3697-82 | 1:200 |  |
| LAG3 | BUV805 | BD Biosciences | 748540 | 1:100 |  |
| LAG3 | BV605 | BD Biosciences | 745214 | 1:100 |  |
| LY6G | BV711 | BD Biosciences | 563979 | 1:200 |  |
| LY6C | | BV605 | BD Biosciences | 563011 | 1:200 |
| MHC-II | BUV737 | Invitrogen | 14-5321-81 | 1:500 |  |
| NK1.1 | FITC | Biolegend | 108706 | 1:200 |  |
| PD-1 | BV785 | Biolegend | 135225 | 1:200 |  |
| PD-L1 | BUV395 | BD Biosciences | 568308 | 1:200 |  |

**Supplemental Table 4: Immunohistochemistry and Immunofluorescence Antibodies**

| **Antibody** | **Company** | **Catalogue number** | **Dilution** | **Incubation** |
| --- | --- | --- | --- | --- |
| CD45 | Abcam | ab550539 | 1:100 | 4°C overnight |
| CK8/CK18 | Abcam | ab53280 | 1:250 | 1hr RT |
| CD19 | Abcam | Ab245235 | 1:500 | 1hr RT |
| AF594 goat anti-rabbit | ThermoFisher | a11037 | 1:300 | 1hr RT |
